# Simplicity, skills, and pitfalls of ascending aortic cannulation for type A aortic dissection

**DOI:** 10.1186/1749-8090-8-161

**Published:** 2013-06-26

**Authors:** Shinichi Taguchi, Atsuo Mori, Ryo Suzuki, Osamu Ishida

**Affiliations:** 1 Department of Cardiovascular Surgery, Kawasaki Municipal Hospital, 12-1 Shinkawadori, Kawasaki-ku, Kawasaki-city, Kanagawa 210-0013, Japan

**Keywords:** Ascending aortic cannulation, Type A aortic dissection, Seldinger technique, Skills and pitfalls

## Abstract

**Background:**

Ascending aortic cannulation for an antegrade central perfusion during surgery for type A aortic dissection is simple and can be performed within median sternotomy. This cannulation is performed routinely without problems in our hospital. Using our experience, the skills and pitfalls were clarified to make this challenging procedure successful.

**Methods:**

29 cases of ascending aortic cannulation using the Seldinger technique for insertion were studied. All insertions were performed with the guidance of transesophageal echocardiography alone. The cannulas were inserted after decompressing the aorta by initiating cardiopulmonary bypass with femoral artery cannulation. From our experience, the skills required for this procedure are the abilities to carefully assess the needle insertion site preoperatively, sense resistance to needle insertion twice, and ensure the guide wire is in the descending aorta and distal arch. The pitfalls are entrance of the guide wire into the false lumen and dilatation of the false lumen during the insertion procedure.

**Results:**

There were no complications associated with ascending aortic cannulation. Regarding morbidity, 2 cases of brain infarction occurred. There were 3 hospital deaths unrelated to the procedure.

**Conclusions:**

In surgery for type A aortic dissection, ascending aortic cannulation using the Seldinger technique is simple to perform. We found that some practical skills and precautions were required to make this procedure successful.

## Background

During surgery for type A aortic dissection, the perfusion route is of great interest to improve the outcome of the procedure [1]. There are several choices of route, which include femoral artery, axillary artery, ascending aorta, and left ventricular apex. Among these, antegrade central perfusion using the ascending aorta is simple and can be performed within median sternotomy. This cannulation is performed routinely without problems at our hospital. To make this challenging technique successful and simple, we clarify some of the skills required and pitfalls we found.

## Methods

During the period of 5 years from July, 2007 to June 2012, 44 aortic dissection operations were performed at our hospital. 42 operations were for type A aortic dissection and 2 for type B. Among the 42 operations for type A, 33 were performed using ascending aortic cannulation, 5 using right axillary artery cannulation, and 4 using femoral artery cannulation alone. Among the 33 operations using ascending aortic cannulation, 29 were performed using the Seldinger technique for inserting 16, 18, or 20Fr heparin-coated flexible thin-walled cannulas (Fem Flex-II cannulas: Edwards Life Sciences Research Medical, Midvale, Utah, USA) into the true lumen. The 4 operations using ascending aortic cannulation without the Seldinger technique were performed using normal right-angled cannulas through a small stab wound in the aorta. These were cases with thin and thrombosed false lumen. The 29 operations using the Seldinger technique were studied. 28 of the 29 cases were performed under true lumen cannulations. In 1 case with a narrow true lumen and a large entry in the ascending aorta, only false lumen cannulation could be obtained.

Patient profiles, DeBakey types, and operative procedures are summarized in Table 1. Informed consent for the procedure was obtained.

**Table 1:** Patient profiles and methods

**Number**	**29**
Age	58.2 ± 13.2 years
Gender	18 males; 11 females
Indications	
DeBakery type I	16
type II	4
type III	9
Operations	
Ascending aortic replacement	21
Ascending, and arch replacement	5(1)
Root, and ascending aortic replacement	1
Root, ascending, and arch replacement	2(2)

Age is expressed as mean ± standard deviation.

Parentheses indicates the number of operations with concomitant right coronary artery bypass grafting.

The Seldinger technique was performed after initiating a cardiopulmonary bypass with a femoral artery cannula and a two-stage cannula inserted into the right atrium. Applying this cardiopulmonary bypass, the aorta was decompressed to avoid disrupting the ascending aorta during the Seldinger technique and when inserting the ascending aortic cannulas. All insertions using this technique were performed with the guidance only of transesophageal echocardiography (TEE). Descending aorta and distal arch images were obtained to visualize the guide wire in the true lumen. Distal arch images were also obtained to visualize placement of cannula tips and color Doppler flow inside the true lumen.

**Figure 1:**
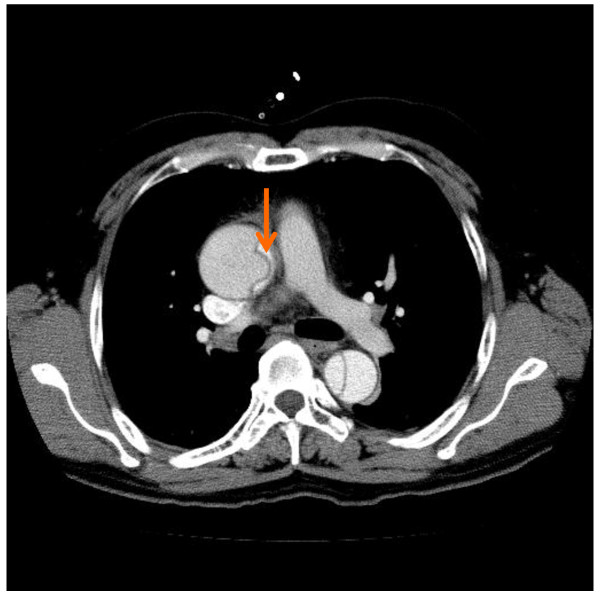
**Typical preoperative computed tomography.** The arrow indicates the estimated direction of needle insertion into the true lumen.

Skills and pitfalls of the technique are discussed together with methods in detail:

1) Point of needle insertion should be precisely assessed preoperatively by studying computed tomography (CT). Usually, the true lumen is at the left side of the ascending aorta, which is the inner curvature (Figure 1).

Because the needle insertion site of the ascending aorta is difficult to visualize with TEE, preoperative enhanced CT images are useful to assess the location and depth of the true lumen. If the true lumen is narrow with a circular dissection, it is better to use another cannulation technique. In emergent cases, surgeons discuss cannulation preoperatively; otherwise, surgeons, anesthetists, perfusionists, and nurses discuss it in a preoperative conference while studying CT images.

**Figure 2:**
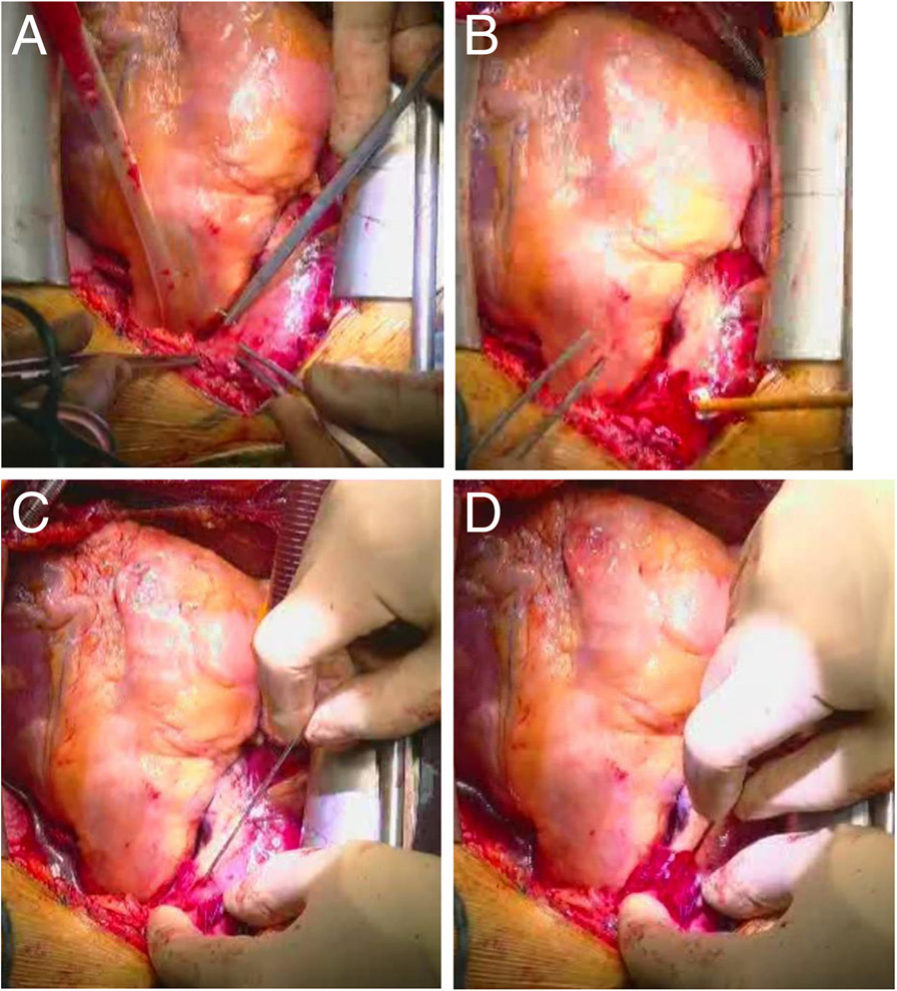
**The bottom parts of the photographs indicate the direction of the patient’s head.** (**A**) Stitches are placed for a single purse string suture in the ascending aorta. (**B**) A tourniquet to snare the cannula is applied. (**C**,**D**) For needle insertion, it is important to sense resistance twice. (**C**) First, at the outer wall of the false lumen. (**D**) Second, at the intimal flap.

**Figure 3:**
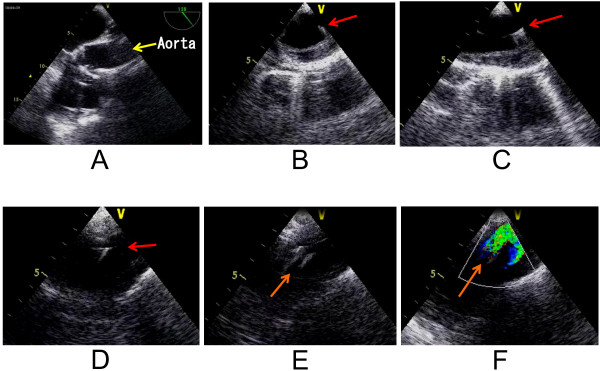
**Images of transesophageal echocardiography.** (**A**) Needle insertion is difficult to visualize in the ascending aorta. (**B**,**C**,**D**) Guide wire (arrows) in the true lumen. (**B**) Short axis of descending thoracic aorta. (**C**) Long axis of descending thoracic aorta. (**D**) Distal aortic arch. (**E**) Cannula tip (arrow) in the distal aortic arch. (**F**) Color Doppler flow through the cannula forms a U-turn shape. Arrow indicates direction of perfusion through the cannula.

**Figure 4:**
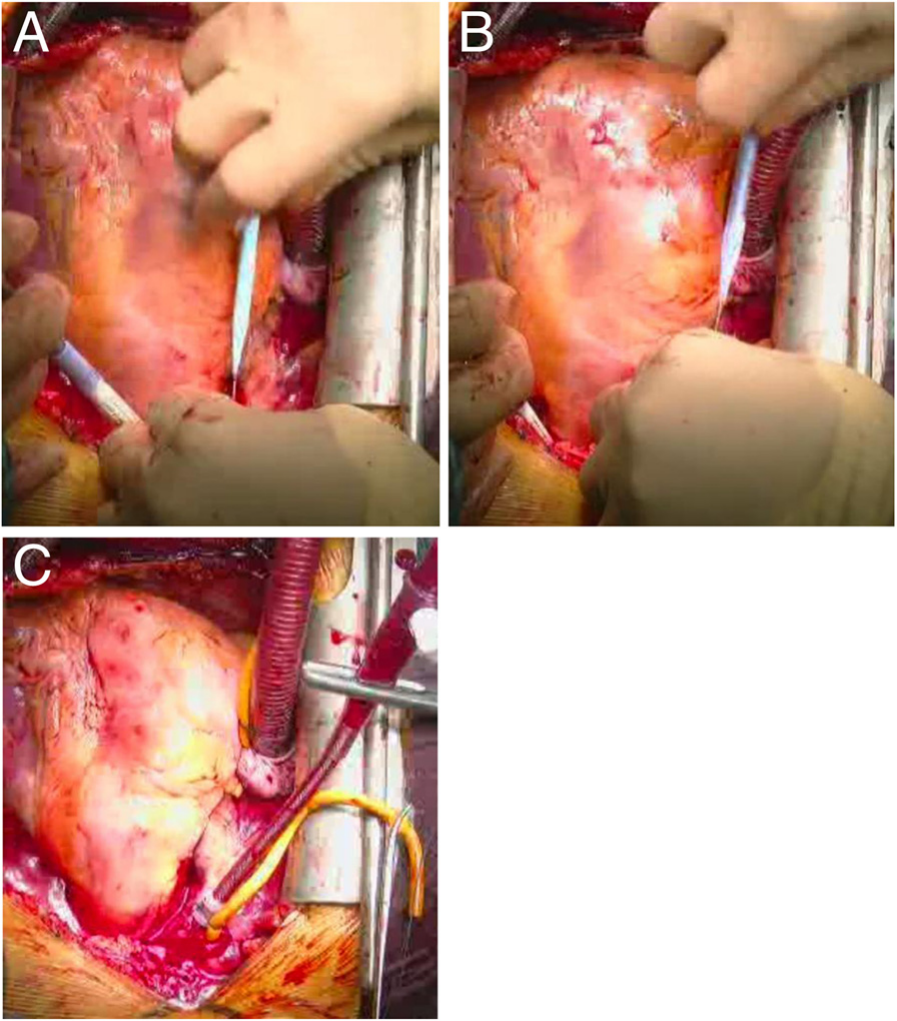
**The bottom parts of the photographs indicate the direction of the patient’s head.** (**A**) Dilator is inserted through the guide wire. 3 dilators of different sizes are used for gradual dilatation. (**B**) Fem Flex-II cannula is inserted through the guide wire. (**C**) The cannula is secured at an optimal depth.

**Figure 5:**
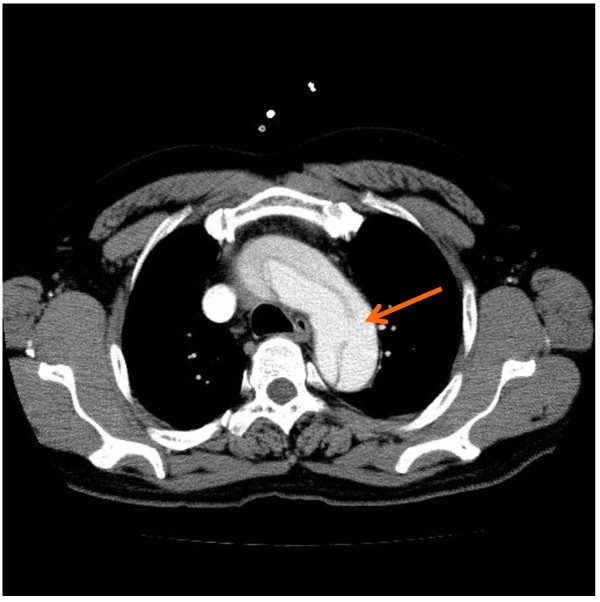
**In the same patient as in Figure **1**, the guide wire was inserted into the false lumen of the descending aorta through the entry (arrow).**

**Figure 6:**
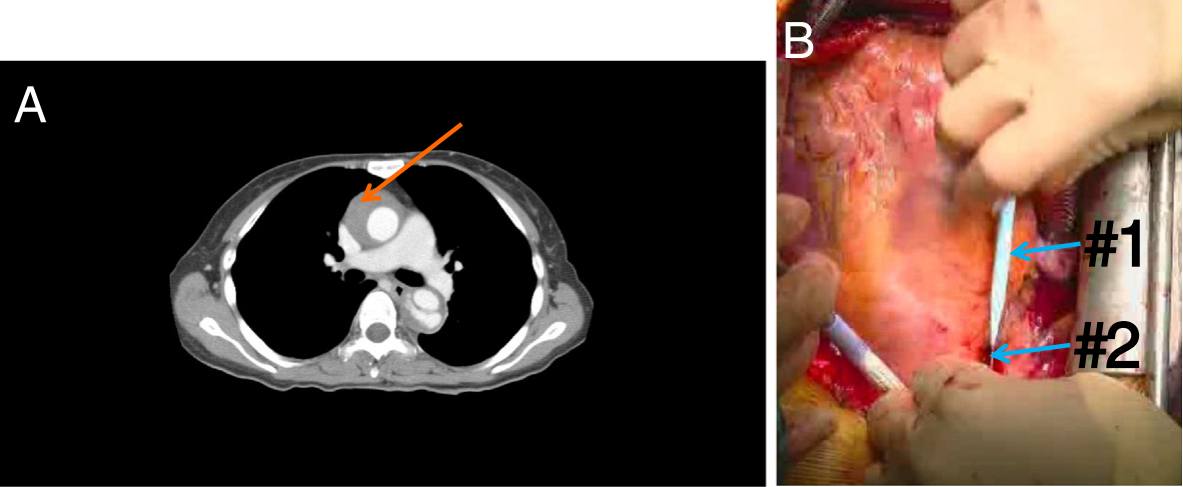
**(A) A case of thrombosed ascending aortic false lumen (arrow) with retrograde aortic dissection of DeBakey type III, in which the false lumen dilated when performing the Seldinger technique.** (**B**) Dilatation was estimated to be the result of formation of a small entry due to the difference in diameter between dilator (#1) and guide wire (#2). The bottom part of the photograph indicates the direction of the patient’s head.

2) Before right atrial cannulation, a single purse string suture was applied to the outer wall of the false lumen of the ascending aorta (Figure 2-A). The stitches were placed deeply to enter the false lumen completely. Although the outer wall seems to be very thin, it usually tolerates sutures. A tourniquet is applied to snare the cannula in the usual manner (Figure 2-B).After cannulating the right atrium and initiating cardiopulmonary bypass, the Seldinger technique is performed. When inserting the needle, it enters the false lumen first and then the true lumen via the intimal flap. For true lumen perfusion, it is important to sense resistance twice; first at the outer wall of the false lumen (Figure 2-C), and second at the intimal flap (Figure 2-D). In most cases, needle insertion trial is needed only once to obtain the true lumen. Only a few cases in this study needed the needle to be inserted more than once because the guide wire was introduced into the false lumen. Usually, TEE does not visualize images of the ascending aorta where the needle is inserted (Figure 3-A).

3) Next, insert the guide wire through the needle. Ensure the guide wire is in the true lumen of the descending aorta (Figure 3-B,C) and the distal arch (Figure 3-D) before dilating the cannulation site. Apply dilators to dilate the outer wall and the intimal flap. 3 dilators of different sizes are applied gradually to dilate them (Figure 4-A). Insert the aortic cannula through the guide wire (Figure 4-B). The tip of the cannula usually enters the true lumen of the aortic arch and can be visualized with TEE (Figure 3-E). Secure the cannula (Figure 4-C), and start ascending aortic perfusion. Blood flow through the cannula in the aortic arch can be visualized as a U-turn flow (Figure 3-F) due to the flow from the femoral arterial perfusion.

4) In a case of retrograde ascending aortic dissection of DeBakey type III, the guide wire was inserted from the true lumen into the false lumen of the descending aorta through the entry (Figure 5). In such a case, it is important not to insert the cannula too deeply for true lumen perfusion.

5) In a case of thrombosed ascending aortic false lumen in retrograde aortic dissection of DeBakey type III (Figure 6-A), the false lumen dilated when only the guide wire was inserted. At this time, a small entry is constructed in the intimal flap for application of dilators. This small entry can induce dilatation of the false lumen. Entry formation results from the difference in diameter between dilator and guide wire (Figure 6-B).

## Results

Table 2 summarizes the results. There were no complications associated with ascending aortic cannulations. Regarding morbidity, there were 2 cases of brain infarctions. Regarding mortality, there were 3 cases. 2 were operative deaths: one due to hemorrhage with low output syndrome and the other due to hemorrhage. Both died on the 1^st^ post-operative day (POD). One patient died due to brain infarction and mediastinitis on 34^th^ POD.

**Table 2:** Results

**Complications associated with ascending aortic cannulations**		**None**
Morbidity		2(brain infarctions)
Hospital deaths (Operative deaths)		3(2)
1 case of ascending aortic replacement	hemorrhage, LOS	1 POD
1 case of root, ascending, and arch replacement	hemorrhage	1 POD
1 case of ascending aortic replacement	brain infarction, mediastinitis	34 POD

LOS: low output syndrome, POD: post-operative day.

## Discussion

Ascending aortic cannulation in type A aortic dissection has been used for a long time mainly to backup arterial inflow or as a bail-out procedure [2,3]. In 2003, routine ascending aortic cannulation aimed at true lumen perfusion was reported [4]. To ensure true lumen cannulation, the Seldinger technique was described by Zapolanski in 2004 as a letter [5]. In 2007, Reece et al. [6] and Inoue et al. [2] published papers on using this technique in 24 cases and 32 cases respectively. Rapid establishment of antegrade systemic perfusion and simplicity of the procedure were insisted.

The merits of this procedure in contrast to femoral artery perfusion are to prevent malperfusion and retrograde cerebral embolism [3]. Merits in contrast to axillary artery perfusion are ease of induction in hemodynamic instability, and effectiveness in dissections extending into the innominate artery [7]. Demerits of this procedure are possible risk of disrupting cannulation site, thromboembolism, and extension of dissection [3].

We used ascending aortic perfusion together with femoral artery cannulation. The objective of femoral perfusion was to decompress the ascending aorta, which counteracts the possible risk of disruption, i.e., the demerit of ascending aortic cannulation. It has been discussed that femoral perfusion alters the arrangement of the true and false lumen in the ascending aorta [8], which was denied by Inoue from epiaortic ultrasound scan (EPI) observations [8]. The demerit of using femoral perfusion, which is retrograde cerebral infarction does not arise frequently in acute dissections as Kamiya et al. states [9]. Overall, we consider that the merits of simultaneously using femoral artery perfusion override the demerits. Another demerit of femoral perfusion is malperfusion, which can be overcome using TEE to detect superior mesenteric artery malperfusion [1], which we plan as the next step.

As described in methods, not all of our procedures were performed with ascending aortic cannulation, although most were. We have postulated the use of right axillary artery cannulation in cases of anticipated arch replacement under hemodynamically stable conditions, and circular dissection with a narrow true lumen, and in cases when it is necessary to prevent malperfusion to the brain during shock or cardiac tamponade. We have postulated the sole use of femoral artery cannulation as in cases of hemodynamic instability due to ascending aortic rupture. Ascending aortic cannulations performed without the Seldinger technique were mostly cases of DeBakey type II with thin and thrombosed false lumen.

As mentioned, we performed the procedure with TEE guidance alone. This contradicts Inoue et al., who insisted on the use of EPI guidance [3]. In our series, we made the procedure as simple as possible, which omitted EPI guidance. Although our series comprised only 29 cases, there were no complications that seemed to be associated with this strategy.

As Saleh et al. wrote in a letter, combining TEE and EPI provides more information [8]. In reply to this letter, Inoue stated that information on the transverse arch and descending aorta obtained by TEE is less important than information obtained by EPI on the ascending aorta and arch [3,8]. We think that for a stable true lumen perfusion, the ascending aortic cannula must be inserted deep enough to place the tip in the aortic arch, especially in short ascending aortic cases. The cannula tip in the aortic arch is also shown by Reece et al. in their figure [6]. The position of the cannula shown in Figure 3-E is usually observed during this procedure, where TEE provides sufficient information. The flow images Figure 3-F shows are in the distal arch, which is better visualized with TEE than EPI. We concede that a disadvantage of not using EPI is in confidently ensuring the first needle insertion. However, as far as the cannula tip or the blood flow from the cannula is visualized in the true lumen, safe perfusion can be obtained. Of course, concomitant use of EPI is desirable to obtain more information.

A case of thrombosed false lumen, which was dilated during the procedure, was performed completely in contradiction to Reece et al., who postulated this situation as a contraindication [6]. The reason was the risk of embolus with cannulation through thrombosed false lumen or intramural hematoma. After experiencing dilatation of the false lumen, we also think this contraindication is due to the possibility of complications associated with false lumen dilatation.

Tiwari et al. reviewed literature, comparing ascending aortic cannulation and peripheral arterial cannulation in acute type A aortic dissection [10]. They found that central cannulation has a lower mortality rate and a lower incidence of malperfusion, but a higher stroke rate.

Although our series had 2 cases of brain infarctions, we could not assess whether these complications could be prevented or not using other cannulations. At least, we do not think the 2 cases were due to retrograde perfusion via femoral artery performed together with ascending aortic perfusion. The patient who survived had an aberrant left vertebral artery originating directly from the arch which probably led to malperfusion. The patient who died was a 37 year-old male who showed no signs of atherosclerosis observed by TEE or CT. As stated by Kamiya et al., patients with acute dissection of the ascending aorta rarely manifest atherosclerosis or plaque in the descending aorta [9].

From their experience, Kamiya et al. compared ascending aorta and femoral artery cannulation for acute aortic dissection type A [9]. Although mortality was lower in the aortic cannulation group, the difference was not statistically significant. They stated that femoral artery cannulation is not so inferior to axillary artery cannulation. Our series of cannulations at sites other than the ascending aorta includes a very small number for comparing cannulation sites.

## Conclusions

We have evaluated our experience of ascending aortic cannulation using the Seldinger technique for type A aortic dissection operations, which is simple. The skills required for this procedure are careful assessment of needle insertion site, sensing resistance to needle insertion twice, and ensuring that the guide wire is in the descending aorta and the distal arch. The pitfalls are reentrance of the guide wire through the entry to the false lumen and dilatation of the false lumen in cases of thrombosed false lumen.

## Competing interests

The authors declare that they have no competing interests.

## Authors’ contributions

ST was the chief and director of the department, and introduced the technique described in the manuscript to the department. All authors contributed to operations and care of patients. All authors read and approved the final manuscript.
